# C-Cell Hyperplasia and Cystic Papillary Thyroid Carcinoma in a Patient with Type 1B Pseudohypoparathyroidism and Hypercalcitoninaemia: Case Report and Review of the Literature

**DOI:** 10.3390/jcm12247525

**Published:** 2023-12-06

**Authors:** Davide Ferrari, Carla Pandozzi, Alessia Filice, Christopher Nardi, Alessia Cozzolino, Rossella Melcarne, Laura Giacomelli, Marco Biffoni, Cira Di Gioia, Elisabetta Merenda, Giulia Del Sindaco, Angela Pagnano, Riccardo Pofi, Elisa Giannetta

**Affiliations:** 1 Department of Experimental Medicine, Sapienza University of Rome, Viale Regina Elena 324, 00161 Rome, Italy; davide.ferrari@uniroma1.it (D.F.); carla.pandozzi@uniroma1.it (C.P.); alessia.filice@uniroma1.it (A.F.); christopher.nardi@uniroma1.it (C.N.); alessia.cozzolino@uniroma1.it (A.C.); elisa.giannetta@uniroma1.it (E.G.); 2 Department of Translational and Precision Medicine, Sapienza University of Rome, 00185 Rome, Italy; rossella.melcarne@uniroma1.it; 3 Department of General and Specialized Surgery, Sapienza University of Rome, 00185 Rome, Italy; laura.giacomelli@uniroma1.it (L.G.); marco.biffoni@uniroma1.it (M.B.); 4 Department of Radiological, Oncological and Pathological Sciences, Sapienza University of Rome, 00185 Rome, Italy; cira.digioia@uniroma1.it (C.D.G.); elisabetta.merenda@uniroma1.it (E.M.); 5 Endocrinology Unit, Fondazione IRCCS Ca’ Granda Ospedale Maggiore Policlinico, 20122 Milan, Italy; giulia.delsindaco@gmail.com (G.D.S.); angela.pagnano@unimi.it (A.P.); 6 Department of Clinical Sciences and Community Health, University of Milan, 20122 Milan, Italy; 7 Oxford Centre for Diabetes, Endocrinology and Metabolism, NIHR Oxford Biomedical Research Centre, University of Oxford, Oxford OX1 2JD, UK

**Keywords:** pseudohypoparathyroidism, hypercalcitoninaemia, C-cell hyperplasia, papillary thyroid cancer, calcitonin

## Abstract

Hypercalcitoninaemia has been described in patients with pseudohypoparathyroidism (PHP) type 1A and 1B. Elevated calcitonin levels are thought to result from impaired Gsα receptor signaling, leading to multiple hormone resistance. Evidence on the risk of medullary thyroid carcinoma (MTC) or C-cell hyperplasia in PHP patients with hypercalcitoninaemia is lacking. A 43-year-old Caucasian man was referred to our endocrinology clinic for chronic hypocalcemia associated with elevated serum parathormone levels and a single cystic thyroid nodule. The patient did not show skeletal deformities, and screening for concomitant hormone resistances was negative, except for the presence of elevated serum calcitonin levels. The workup led to a molecular diagnosis of sporadic PHP1B. Fine needle aspiration of the thyroid nodule was not diagnostic. The calcium stimulation test yielded an abnormal calcitonin response. Given the scarcity of data on the risk of thyroid malignancy in PHP and calcium stimulation test results, total thyroidectomy was performed. Histological examination revealed cystic papillary thyroid cancer in a background of diffuse C-cell hyperplasia. To our knowledge, we are the first to describe a rare form of thyroid cancer combined with C-cell hyperplasia in a patient with PHP and hypercalcitoninaemia. In the present case, a mere receptor resistance might not fully explain the elevated calcitonin levels, suggesting that hypercalcitoninaemia should be carefully evaluated in PHP patients, especially in the case of concomitant thyroid nodules. Further studies on larger cohorts are needed to elucidate this topic.

## 1. Introduction

Pseudohypoparathyroidism (PHP) is a group of rare endocrine disorders of calcium metabolism characterized by renal resistance to the parathyroid hormone (PTH) due to impaired intracytoplasmic receptor signaling caused by de novo or autosomal dominant (epi)genetic mutations of *GNAS* locus. Five variants of PHP have currently been described: type 1A, 1B, 1C, 2, and pseudo-PHP (PPHP). PHP-1A is the most common variant, accounting for 70% of total cases reported. The genotype differences in PHP variants reflect a heterogeneity of phenotypes that generally share the biochemical features of hypoparathyroidism, namely hypocalcemia and hyperphosphatemia, but with an unexpected elevation of serum PTH. Another common feature of PHP (especially concerning PHP-1A and 1C) is the presence of concomitant hormone resistances involving thyroid stimulating hormone (TSH), gonadotropins, calcitonin, and growth hormone (GH) release peptide (GHRH). Moreover, PHP can manifest with a spectrum of several anomalies (short stature with stocky habitus, round face, brachydactyly, ectopic subcutaneous ossifications, dental anomalies, and intellectual disability) known as Albright Hereditary Osteodystrophy (AHO) [1,2], especially in PHP1A, PHP1C, and PPHP variants [3]. Conversely, PHP-1B patients generally show PTH resistance and, in 60–70% of cases, TSH resistance without AHO features and other hormone-signaling disorders. In contrast to other variants, pseudo-PHP is not associated with resistance to PTH or other hormonal resistances despite displaying evident AHO features.

The complex *GNAS* gene on chromosome 20q13.3 encodes for several products, including the stimulatory guanine (Gsα) that links the membrane G-protein coupled receptor (GPCR) to the intracytoplasmic cAMP/PKA pathway, mediating the cellular effects of different hormones including PTH, TSH, gonadotropins, calcitonin, and GHRH. *GNAS* is an imprinted locus and maternal imprinting is prevalent in renal proximal tube, thyroid gland, pituitary, and ovarian cells. Therefore, the phenotypical result of *GNAS* mutations is strictly dependent on its parental origin. In brief, while PHP1A is generally caused by inherited or de novo (with similar incidences) inactivating mutations of the maternal allele of *GNAS*, PHP1B is characterized by defects in DNA methylation that can be maternally inherited (AD-PHP1B, 10–20%) [4] or sporadic. On the other hand, when *GNAS* mutations occur in the paternal allele, they generally cause PPHP. Regarding PHP1C, mutations in the *GNAS* locus have been rarely described [5] and its exact etiology remains unknown. A deeper walkthrough on the molecular background of PHP was discussed in several other articles [6,7,8].

Hypercalcitoninaemia can be observed in PHP patients [9,10,11,12], with or without other hormone resistances, and a positive response to pentagastrin and calcium stimulation tests [9,12,13] has been reported. The exact cause and consequences of elevated serum calcitonin levels in patients with PHP have not been elucidated yet, albeit a hormone resistance mechanism resulting from impaired Gsα receptor signaling has been speculated [14]. The scenario becomes even more complicated when one or multiple thyroid nodules are found in the clinical context of PHP and hypercalcitoninaemia.

Within the vast series of thyroid nodules, malignancy represents only 5% of all cases. The real diagnostic challenge is to identify malignant nodules among the more prevalent benign nodular conditions. Papillary thyroid carcinoma (PTC) is the most common histology of thyroid cancer, accounting for approximately 85–90% of thyroid cancers. Typically, PTC presents as a solid nodule on ultrasound imaging [15]. Conversely, cystic nodules represent a less common presentation of PTC, occurring in fewer than 10% of cases. Although the finding of cystic thyroid formation may be related to a risk of malignancy of less than 5%, the malignant potential of these nodules should never be overlooked. The diagnosis of PTC is primarily based on the typical cytological and histological features, but in some cases of histologic uncertainty, immunohistochemical stains, such as HBME-1, may be used to help classify unusual presentations of PTC [16]. To the best of our knowledge, no cases of PTC in patients with PHP have been described in the literature so far.

Based on these initial reflections, we present a case of concomitant hypercalcitoninaemia and solitary cystic thyroid nodule in a patient diagnosed with PHP1B, aiming to further increase the existing yet limited evidence on this rare genetic disease.

## 2. Case Presentation

**Figure 1:**
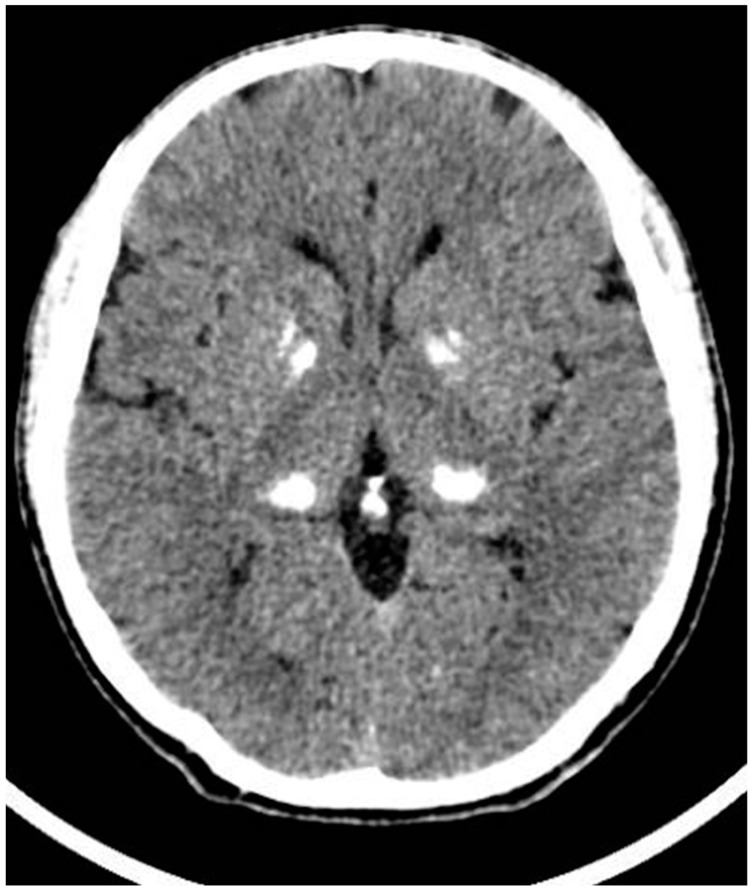
Fahr syndrome. CT scan showing bilateral calcifications of the basal ganglia, particularly present in the dorsal thalamus, globus pallidus, and putamen areas.

A 43-year-old Caucasian man presented at our endocrinology clinic for follow-up of a solitary thyroid nodule. The patient’s laboratory records revealed a history of chronic hypocalcemia (with mean serum calcium levels of 6.5 mg/dL = 1.63 mmol/L) and hyperphosphatemia, which were observed since the age of 30 years but were neither investigated nor treated thereafter. The patient presented with good clinical conditions, alert and collaborating. Physical examination did not show skeletal abnormalities. The patient reported headache, nocturnal cramps, and occasional paraesthesias; however, no signs of tetany were described. The biochemical analyses performed at our laboratory confirmed low serum calcium (6.5 mg/dL = 1.63 mmol/L) and high phosphorus (5.6 mg/dL = 1.81 mmol/L) levels. Surprisingly, serum PTH levels were high (220 pg/mL, 3.8 × ULN). The patient showed suboptimal levels of vitamin D (25OHD 26 ng/mL), and 24 h urine analysis showed low calcium excretion (10 mg/24 h) along with normal urinary levels of phosphates, sodium, magnesium, and potassium. In Table 1, all the laboratory assessments performed by the patient are summarized. Creatinine levels were within a normal range (0.81 mg/dL) and celiac disease tests resulted negative. Bone densitometry performed via Dual X-Ray Absorbimetry (DXA) showed good levels of areal bone mineral density (aBMD) of femur neck (0.921 g/cm^2^, Z −0.5), total hip (0.925 g/cm^2^, Z −0.9), and lumbar spine (1.185 g/cm^2^, Z −0.4). A computerized tomography (CT) scan of the head showed diffuse and bilateral calcifications of basal ganglia (dorsal thalami, globus pallidus, and putamen), dentate nuclei, and subcortical white matter of frontal and parietal lobes (Fahr syndrome; Figure 1) [17]. The combination of these clinical and biochemical findings was suggestive for the diagnosis of PHP. Therefore, a molecular analysis to search for *GNAS* locus mutations was performed. The first line NGS test presented negative results. However, the methylation analysis to assess epigenetic defects revealed the presence of multiple alterations in the methylation pattern of the *GNAS* locus and the absence of deletions in the imprinting control region located within the *STX16* gene. Although the presence of an inherited cause could not be definitively excluded, these molecular findings led to the diagnosis of sporadic PHP type 1B. 

Treatment with calcitriol and calcium carbonate was introduced and progressively adjusted based on calcium and phosphorus serum levels up to a stable daily dose of 0.5 μg and 500 mg, respectively. Following the adjustment of calcium (8.4 mg/dL = 2.1 mmol/L) and phosphorus (4.0 mg/dL = 1.3 mmol/L) levels, serum PTH levels continued to be elevated (302 pg/mL, 3.6 times the upper limit of normal). Mineral metabolism abnormalities were searched in the patient’s family (parents and three siblings), and their calcium, phosphorus, and PTH serum levels were found to be within the normal range, supporting the diagnosis of sporadic PHP1B. The patient did not show any AHO features, and TSH, thyroid hormones, gonadotropins, testosterone, GH, and Insulin-like Growth Factor 1 (IGF-1) serum levels were normal, excluding the presence of other hormonal resistances. Given the radiological finding of Fahr Syndrome and the presence of chronic headaches, the patient was referred to a neurology specialist who prescribed amitriptyline therapy. Although thyroid function was found to be normal, calcitonin levels were above the normal range (40.9 ng/mL, 5.2 times the upper limit of normal). The thyroid ultrasound showed a solitary anechoic nodule of 3.8 × 2.2 cm with a solid portion with inhomogeneous echogenicity that was already noticed in previous scans. Doppler ultrasound showed a peripheral and internal vascularity of the nodule. Thyroid parenchyma was characterized by an inhomogeneous signal due to the presence of bilateral hyperechoic striae. No parathyroid glands were detected. Consequently, an ultrasound-guided fine needle aspiration biopsy cytology (FNAB-C) on the nodule was performed. The cytological evaluation showed smears consisting of abundant small colloids mixed with red blood cells and occasional macrophages. Notably, no thyrocytes were observed, shaping the features of a non-diagnostic/cystic result (TIR1C) according to the Italian Reporting System for thyroid cytology [18]. In the workup of suspect medullary thyroid carcinoma (MTC), cytological characteristics can exhibit significant variability, limiting their utility in guiding the preoperative assessment of the patient [19]. To increase sensibility, a calcitonin measurement in fine-needle aspirate washout fluid was performed, obtaining a concentration of 13.8 pg/mL, a value below the internal laboratory cut-off of 20 pg/mL based on other evidence in the literature [20,21,22].

Given the peculiar clinical picture, characterized by an ambiguous finding of hypercalcitoninaemia in the context of a thyroid nodule without a certain diagnosis of benignity and the greater sensitivity and specificity of the measurement of serum calcitonin in the suspicion of a C-cell pathology [23], we decided to perform a calcium stimulation test: 10% calcium gluconate at a dosage of 25 mg/Kg b.w. was administered intravenously at 10 mL/min with serial blood sampling before and, subsequently, after 1, 2, 3, 5, 10, and 15 min following the infusion. The test revealed a baseline calcitonin level of 34.1 pg/mL and a post-infusion maximum calcitonin peak of 651 pg/mL. In agreement with previously reported evidence [24,25], the test was considered positive (Table 1).

**Figure 2:**
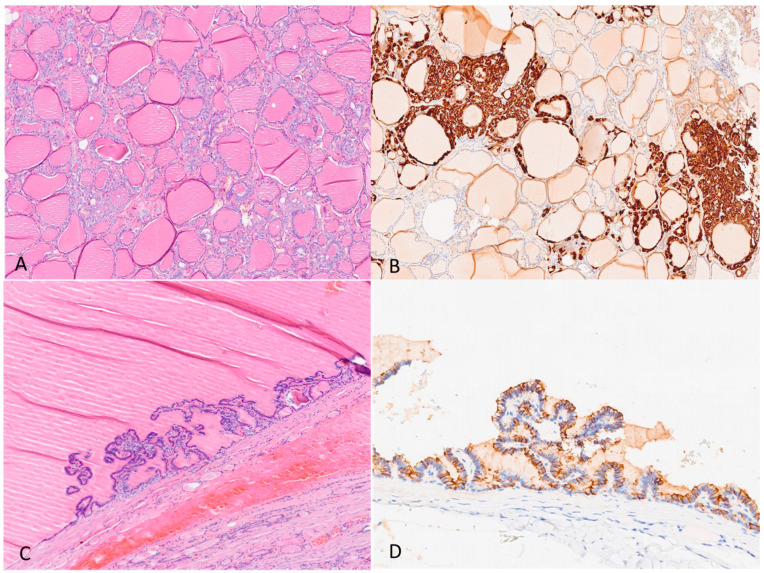
Histological examination of total thyroidectomy showing diffused C-cell hyperplasia and cystic papillary thyroid tumor. (**A**) Hematoxylin and eosin section showing C-cell hyperplasia 2×; (**B**) immunohistochemical staining with anti-calcitonin antibody 4×; (**C**) hematoxylin and eosin sections showing cystic papillary carcinoma 4×; (**D**) immunohistochemical staining with anti-HMBE1 antibody 4×.

Given the results of the calcium gluconate test and the presence of a large cystic nodule extending from the right lobe to the isthmic-paraisthmic location, in agreement with the patient, total thyroidectomy was carried out. The histological examination showed diffuse parafollicular/C-cell hyperplasia (calcitonin+, synaptophysin+, and chromogranin−). Surprisingly, the cystic nodule examination revealed a diagnosis of cystic papillary thyroid carcinoma without lymph node invasion (pT2pN0, R0; 0/15 lymph nodes). This diagnosis was based on the observation of papillary hyperplasia and aspects of incipient neoplastic transformation due to the presence of focal cells with an immunophenotypic profile HMBE-1+, galectin-3−, and CD56−, especially in the context of the epithelial lining of the cyst. No histological evidence of thyroiditis was detected in the rest of the thyroid parenchyma (Figure 2). One left parathyroid gland was surgically removed, revealing no evidence of hyperplasia.

**Figure 3:**
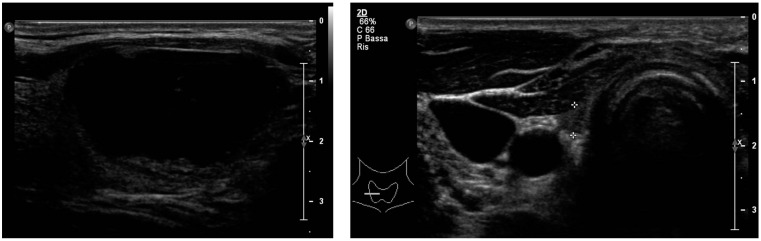

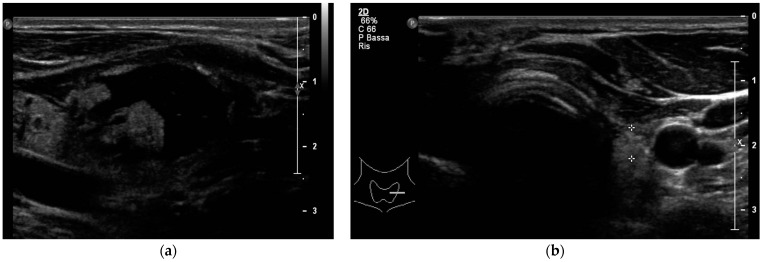
Ultrasound examination of the anterior neck region before (**a**) (with focus on the thyroid nodule) and after (**b**) thyroidectomy (with the “+” symbols delineating the size of the inhomogeneous area in left thyroid lodge attributable to post-surgical remodeling outcomes).

After a multidisciplinary meeting, a surveillance strategy was chosen, and standard levothyroxine suppressive therapy was started. Following surgery, serum PTH decreased to undetectable levels at 1 week, 6 weeks, and 6 months after surgery, and calcium levels experienced a significant drop (9.7 to 7.0 mg/dL), prompting adjustments in medical therapy until stability was achieved. The final regimen comprised twice the pre-surgery dose, with 1000 mg/day of calcium carbonate and 1 μg/day of calcitriol. Table 2 provides a comprehensive overview of the performed follow-up biochemical tests. Post-operative ultrasound scans showed the results of total thyroidectomy with medialization of the great vessels. In the thyroid lodges, there was a non-homogeneous area of 0.5 cm, attributable to the results of post-surgical remodeling. The study of the neck lymph nodes showed only a few lymph node stations with normal appearance (Figure 3). Therefore, a clinical, laboratory, and instrumental follow-up was set up every six months, at least for the first year. A follow-up CT scan was also scheduled to assess the radiological evolution of Fahr Syndrome following surgery and the subsequent drop in PTH levels.

## 3. Discussion

To the best of our knowledge, we are the first research group to describe a case of concomitant cystic papillary thyroid cancer and C-cell hyperplasia in a patient with PHP type 1B and hypercalcitoninaemia. The convergence of these multiple rare conditions in the same patient renders this case particularly intricate in terms of its clinical management. 

**Figure 4:**
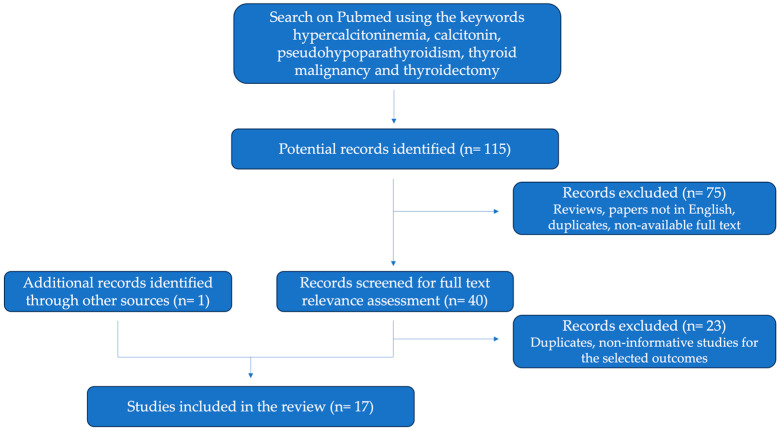
PRISMA flowchart of literature search, determination of eligibility, and inclusion in the present review of literature.

Hypercalcitoninaemia is known to predict with high sensitivity but low specificity a diagnosis of MTC [26]. However, the elevation of calcitonin levels and increased response to stimulation tests were also described in several other conditions, including Hashimoto’s thyroiditis, neuroendocrine neoplasms, papillary thyroid cancer, and cases of concomitant medications such as proton pump inhibitors [27,28]. In order to identify other cases of hypercalcitoninaemia, thyroid cancer, or thyroidectomy in the context of PHP, we performed an extensive search from PubMed and other sources of the literature, using several keywords associated with “pseudohypoparathyroidism” in our algorithm. Namely, the list of used terms included “hypercalcitoninemia”, “calcitonin”, “thyroid malignancy”, and “thyroidectomy”. Records were excluded if published in a language other than English if the full text was not accessible, and if it did not include reports of hypercalcitoninaemia in a PHP context. A total of 116 articles were identified, and 17 were finally selected. Details on the results of our search are available in a PRISMA flowchart (Figure 4). 

Our search revealed that, in the context of PHP, several instances of hypercalcitoninaemia with abnormal responses to stimulation tests have been documented [27,28,29,30]. However, it must be noted that the existing literature is mostly derived from dated sources, with limited relevant articles published after 2010 [13,31,32,33]. Moreover, many of the existing works did not provide a certain molecular diagnosis of PHP, thereby diminishing the reliability of these findings.

Nevertheless, the first instances of hypercalcitoninaemia in PHP patients were initially observed in the 1960s, and various explanations for this phenomenon have been proposed over the last few decades. In 1966, Aliapolious et al. first explored the role of calcitonin in a PHP patient, showing a marked elevation of the hormone concentration within the thyroid gland [34]. These results were followed and confirmed by Lee et al., who described three cases of familial PHP comprising a 28-year-old woman who underwent bilateral subtotal thyroidectomy, and the subsequent analysis revealed the presence of areas with “increased numbers of parafollicular cells” that may resemble a C-cell hyperplasia [35]. Since then, speculations have been made on a possible etiological role of increased calcitonin secretion and accumulation within the thyroid gland on the development of PHP. Conversely, other authors addressed these findings to the longstanding hypocalcemic state that is observed in PHP.

Prompted by the “etiological” hypothesis, in 1968, Suh et al. reported the case of an 11-year-old girl with PHP who was treated with total thyroidectomy. Following surgery, no improvement in hypocalcemia was observed, refuting the possibility of calcitonin’s pivotal role in PHP etiology and on its disease course [36]. Later, in 1973, Deftos et al. investigated calcitonin secretion in patients with hypocalcemic states, including 9 PHP, who were evaluated at baseline and after stimulation tests with pentagastrin or calcium infusion. This study confirmed the higher increase in stimulated calcitonin in PHP patients amongst the four disease groups studied [37]. Given the altered response to calcium stimulation, the authors hypothesized that chronic hypocalcemia may lead to calcitonin accumulation within the thyroid gland and that the hypercalcemic state following calcium infusion stimulation might promote the excessive secretion of accumulated calcitonin. In the same year, Birkenhäger et al. reported the case of a 14-year-old girl with PHP whose baseline calcitonin levels were within the normal range with a significant, but not excessive, response after calcium infusion [38]. Almost a decade later, Wägar et al. described a 33 years old PHP patient with PHP type 1 associated with hypercalcitoninaemia and prolactin and growth hormone deficiency. The patient underwent a pentagastrin stimulation test and a striking high response in calcitonin levels was observed. Subsequently, a thyroid biopsy showed normal thyroid tissue and no malignancies [10]. 

In 1984, Fujii et al. first speculated that hypercalcitoninaemia in PHP may be addressed to peripheral resistance [12]. Ever since, the resistance hypothesis has grown in importance [11] and is currently endorsed by most of the scientific community [13,14].

The reports of hypercalcitoninaemia mainly involved PHP1A patients with other hormonal resistances. In 2001, Vlaeminck-Guillem and colleagues [9] detailed the characteristics of six individuals diagnosed with PHP1A who exhibited elevated levels of both basal and pentagastrin and calcium-stimulated calcitonin. Notably, their study also incorporated a control group that included a PHP1B patient who presented with a normal calcitonin level. The authors ultimately concluded that hypercalcitoninaemia could be considered specific to PHP1A. However, one case of hypercalcitoninaemia in PHP1B was described in 2019 [13], and unpublished but significant observations about several other cases were reported in the 2018 International Consensus Statement on PHP [14].

Amongst the most recently published case reports, Brancatella et al. [33] described a PHP patient with marked AHO features, Fahr syndrome, and hypocalcemia who presented with slightly elevated calcitonin levels despite adequate calcium therapy. 

In 2019, Yavropolou et al. documented the cases of two patients, one with PHP1A and the other one with PHP1B, both presenting with hypercalcitoninaemia [13]. The PHP1A patient also had a concurrent multinodular goiter with a negative FNAB-C result on the larger nodule but a pathological increase in calcitonin levels following the pentagastrin infusion test. Consequently, and in agreement with the patient, a total thyroidectomy was performed. However, the report lacked details on the histological examination, only mentioning the absence of thyroid malignancy. In contrast, the PHP1B patient had a normal thyroid gland without evidence of thyroid nodules and did not undergo surgery. 

In 2018, Underbjerg et al. evaluated 62 patients with non-surgical hypoparathyroidism and 31 with PHP, reporting higher calcitonin levels in the latter group [32]. This study provided one of the largest assessments of calcitonin levels in PHP, strongly demonstrating its impairment. 

In a recent semi-structured survey [39] conducted across five Italian centers, data on the clinical management of PHP were collected. The findings from this study indicate a notable lack of investigation into calcitonin levels, highlighting the necessity for increased attention to this aspect. 

While the majority have elucidated the rise in calcitonin levels as a consequence of Gsα signaling disruption [13,14], the concomitant presence of one or more thyroid nodules further deepens the complexity of clinical management since data on the risk of MTC in patients with PHP are currently lacking. Among the previously described cases, the subset of patients who underwent thyroidectomy showed negative histological examination with no signs of thyroid malignancy [10,13]. So far, no published evidence indicates that hypercalcitoninaemia leads to a significant risk of MTC or significant C-cell hyperplasia, as well as any other form of thyroid cancer in PHP patients [14]. Indeed, no records were found by our comprehensive literature search using the keywords “thyroid malignancy” and “pseudohypoparathyroidism”. These results were confirmed even after incorporating the search by adding the keywords “thyroid cancer” and “thyroid carcinoma”.

In this scenario, our case is noteworthy as it represents the first documented instance of thyroid cancer and C-cell hyperplasia in a patient with PHP1B and hypercalcitoninaemia. Moreover, to our knowledge, this is also the first report of calcium gluconate employment to evaluate stimulated calcitonin response in the context of PHP. The exact cause leading to hypercalcitoninaemia in our patient remains unclear. Calcitonin resistance due to the impairment of Gsα signaling might certainly play a pivotal role. However, the presence of C-cell hyperplasia suggests that the mechanisms contributing to the development of hypercalcitoninaemia in PHP could extend beyond a mere derangement in second messengers’ pathways. 

In this context, the concomitant finding of cystic papillary thyroid cancer further makes this case distinctive. Indeed, the cystic variant of PTC is rare. Ultrasound is the first-level examination for the study of thyroid nodules, but the FNAB-C is the gold standard for the evaluation of nodules with suspicious sonographic features [40]. The presence of cystic changes within a thyroid nodule can decrease the accuracy of FNAB-C, given the difficulty of obtaining adequate cellularity. Therefore, diagnosing thyroid cancer by fine-needle aspiration of a lesion with a high cystic content could be very difficult, also due to the degenerative changes often occurring within this subtype of thyroid nodules [41]. Thus, cystic PTC continues to be a diagnostic challenge due to its hypocellular features. Indeed, previous studies have shown that only 0.2% of this histotype has been diagnosed by FNAB-C [42]. Useful clues in the diagnosis can be obtained thanks to the use of specific immunohistochemical stains. PTC is usually reactive to cytokeratins, thyroglobulin, and TTF-1 and negative for synaptophysin and chromogranin. Even galectin-3 and HBME-1 are generally expressed in high proportions, but they are less specific for PTC as they have been found in several subtypes of thyroid cancer. Nonetheless, their expression is not found in normal thyroid tissues, and when present, it seems to suggest a malignant lesion [16]. In terms of clinical management of cystic PTC, the adoption of the conventional guidelines for other well-differentiated thyroid carcinoma is suggested, including possibly post-operative radioactive iodine therapy and the use of levothyroxine suppressive therapy [16]. In the present case, the patient displayed a well-differentiated, early-stage carcinoma in the absence of capsular or lymphatic invasion. Therefore, a surveillance strategy was chosen.

Total thyroidectomy provided a unique opportunity to delve deeper into the intricacies of PHP. While earlier studies have documented parathyroid hyperplasia [43], the available data are limited and often contradictory [44]. In our specific case, no evidence of hyperplastic parathyroid glands was identified during the surgery, and the appearance of the excised parathyroids was normal at the subsequent histological examination. 

Patients with PHP1B manifest varying degrees of PTH-resistant hypocalcemia attributed to distinct genetic mutations associated with the condition [14,45]. This diversity may indicate variations in residual PTH receptor activity. In the present case, calcium levels experienced a significant post-surgery drop, necessitating a twofold increase in calcium and calcitriol therapy compared to the pre-surgery dosage. Simultaneously, PTH levels decreased to undetectable levels and showed no rebound at the last observation six months post-surgery. These findings lead us to speculate that PTH exerted some residual activity in the patient, and the suppression of PTH levels following thyroidectomy resulted in a loss of this residual function, prompting the need for intensified medical therapy.

Notably, histological examination revealed the surgical removal of only one non-hyperplastic parathyroid gland, suggesting the possibility that the remaining glands might be temporarily suppressed via “stunning” mechanisms [46]. Consequently, a potential rebound of PTH levels cannot be excluded, emphasizing the need for periodic follow-up to evaluate potential adjustments in medical therapy.

## 4. Conclusions

This case report and review of the literature are of particular interest due to the lack of similar cases of a rare form of thyroid cancer combined with C-cell hyperplasia in patients with PHP and hypercalcitoninaemia. This paper firstly increases the evidence on disrupted calcitonin feedback in PHP, especially in type 1B. Moreover, to our knowledge, this is the first reported case of cystic papillary thyroid cancer in the context of PHP, indicating that the development of a well-differentiated thyroid tumor deriving from follicular cells might occur in these patients. 

Our study is the first to demonstrate the association of hypercalcitoninaemia with the histological manifestation of diffuse C-cell hyperplasia in PHP1B. Until now, no cases of medullary thyroid carcinoma have been reported in PHP, and hypercalcitoninaemia has been solely attributed to impaired Gsα signaling, supported by the absence of significant related histological changes. The identification of C-cell hyperplasia in our patient prompts us to at least reconsider the notion that hypercalcitoninaemia is always irrelevant in PHP patients. Consequently, we advocate for thyroid ultrasound in all cases exhibiting elevated calcitonin levels. If thyroid nodules are detected, fine-needle aspiration biopsy (FNAB-C) is recommended, and clinical management should adhere to conventional guidelines. A calcitonin stimulation test may be considered to guide subsequent procedures, including thyroid surgery. It must be noted that, in our case, the report of PTC is more likely to be incidental rather than linked to the patient’s condition. Hence, considering the available evidence, the choice to undergo thyroid surgery for patients with PHP, hypercalcitoninaemia, and a thyroid nodule with indeterminate cytology should continue to be based on the physician’s judgment within the framework of a personalized approach. In cases where a conservative approach is chosen, vigilant monitoring is recommended.

Our observations on the significant changes in calcium and PTH levels following thyroidectomy have also provided insights into unanswered questions surrounding PHP. Notably, our experience indicates that a significant decrease in PTH levels following thyroid surgery may result in aggravated hypocalcemia, suggesting a potential residual PTH effect in PHP patients. Hence, we propose careful and periodic monitoring of calcium and PTH levels in patients with PHP undergoing total thyroidectomy.

The exact cause leading to hypercalcitoninaemia in our patient remains unclear and might extend beyond simple hormone resistance, especially considering the concomitant finding of C-cell hyperplasia and PTC. Whether the presence of hypercalcitoninaemia and C-cell hyperplasia is associated with an increased risk of MTC in patients with PHP currently remains an unanswered question, together with the risk of developing other thyroid tumors in this subpopulation of patients. Further studies on larger cohorts are needed to elucidate this new and complex topic, but this case report could be used as input for new research on rapidly evolving topics.

## Figures and Tables

**Table 1:** Results of the calcium gluconate stimulation test.

	T0	T1 (1′)	T2 (2′)	T3 (3′)	T4 (5′)	T5 (10′)	T6 (15′)
Calcitonin (pg/mL)	34.1	651	537	503	392	248	186

**Table 2:** Patient’s biochemical assessment before and after total thyroidectomy.

	*Normal Laboratory Range*	At the Time of Diagnosis (Naive to Therapy)	Before Surgery (after Medical Therapy)	6 Days after Surgery	6 Weeks after Surgery	6 Months after Surgery
PTH, pg/mL	*4–58.1*	220	86.8	<4	<4	<4
Calcitonin, pg/mL	*<10*	34	33.1	-	1.6	<2
Calcium, mg/dL	*8.4–10.2*	6.5	9.7	7.0	8.2	8.5
Phosphorus, mg/dL	*2.5–4.5*	5.6	3.6	4.6	4.1	4.5
Vitamin D, ng/mL	*>30*	26	32.6	-	-	30.0
Creatinine, mg/dL	*0.7–1.3*	0.9	1.0	-	0.9	1.0
TSH, µUI/mL	*0.35–4.94*	1.4	2.64	-	7.08	0.12
fT4, ng/dL	*0.7–1.48*	0.78	-	-	0.82	1.15
Thyroglobulin, ng/mL	*3.68–64.15*					<0.1
Thyroglobulin Antibodies, UI/mL	*<5*					3
LH, mUI/mL	*1.8–8.2*	2.1	-	-	-	-
FSH, mUI/mL	*1.4–9.8*	2.5	-	-	-	-
Testosterone, ng/mL	*3.0–8.1*	6.52	-	-	-	-
ACTH, pg/mL	*3–41*	26	-	-	-	-

## Abbreviations

aBMD	areal Bone Mineral Density
AHO	Albright Hereditary Osteodystrophy
CT	Computerized Tomography
DXA	Dual X-Ray Absorbimetry
FNAB-C	Fine Needle Aspiration Biopsy Cytology
GCPR	G-Protein Coupled Receptor
GH	Growth Hormone
GHRH	Growth Hormone Release Peptide
MTC	Medullary Thyroid Carcinoma
PHP	Pseudohypoparathyroidism
PPHP	Pseudo-pseudohypoparathyroidism
PTC	Papillary Thyroid Carcinoma
PTH	Parathyroid Hormone
TSH	Thyroid-Stimulating Hormone

## References

[1] McMullan P., Germain-Lee E.L. (2022). Aberrant Bone Regulation in Albright Hereditary Osteodystrophy dueto Gnas Inactivation: Mechanisms and Translational Implications. Curr. Osteoporos. Rep..

[2] Albright F., Forbes A.P., Henneman P.H. (1952). Pseudo-pseudohypoparathyroidism. Trans. Assoc. Am. Physicians.

[3] Mantovani G., Linglart A., Garin I., Silve C., Elli F.M., de Nanclares G.P. (2013). Clinical utility gene card for: Pseudohypoparathyroidism. Eur. J. Hum. Genet..

[4] Elli F.M., Linglart A., Garin I., de Sanctis L., Bordogna P., Grybek V., Pereda A., Giachero F., Verrua E., Hanna P. (2016). The Prevalence of GNAS Deficiency-Related Diseases in a Large Cohort of Patients Characterized by the EuroPHP Network. J. Clin. Endocrinol. Metab..

[5] Thiele S., de Sanctis L., Werner R., Grotzinger J., Aydin C., Juppner H., Bastepe M., Hiort O. (2011). Functional characterization of GNAS mutations found in patients with pseudohypoparathyroidism type Ic defines a new subgroup of pseudohypoparathyroidism affecting selectively Gsalpha-receptor interaction. Hum. Mutat..

[6] Weinstein L.S. (2001). The stimulatory G protein alpha-subunit gene: Mutations and imprinting lead to complex phenotypes. J. Clin. Endocrinol. Metab..

[7] Juppner H. (2021). Molecular Definition of Pseudohypoparathyroidism Variants. J. Clin. Endocrinol. Metab..

[8] Tafaj O., Juppner H. (2017). Pseudohypoparathyroidism: One gene, several syndromes. J. Endocrinol. Investig..

[9] Vlaeminck-Guillem V., D'Herbomez M., Pigny P., Fayard A., Bauters C., Decoulx M., Wemeau J.L. (2001). Pseudohypoparathyroidism Ia and hypercalcitoninemia. J. Clin. Endocrinol. Metab..

[10] Wagar G., Lehtivuori J., Salven I., Backman R., Sivula A. (1980). Pseudohypoparathyroidism associated with hypercalcitoninaemia. Acta Endocrinol..

[11] Kageyama Y., Kawamura J., Ajisawa A., Yamada T., Iikuni K. (1988). A case of pseudohypoparathyroidism type 1 associated with gonadotropin resistance and hypercalcitoninaemia. Jpn. J. Med..

[12] Fujii H., Higashi K., Morita M., Sato T. (1984). A case of pseudohypoparathyroidism (PHP) associated with multiple hormonal abnormalities. Jpn. J. Med..

[13] Yavropoulou M.P., Chronopoulos E., Trovas G., Avramidis E., Elli F.M., Mantovani G., Zebekakis P., Yovos J.G. (2019). Hypercalcitoninaemia in pseudohypo-parathyroidism type 1A and type 1B. Endocrinol. Diabetes Metab. Case Rep..

[14] Mantovani G., Bastepe M., Monk D., de Sanctis L., Thiele S., Usardi A., Ahmed S.F., Bufo R., Choplin T., De Filippo G. (2018). Diagnosis and management of pseudohypoparathyroidism and related disorders: First international Consensus Statement. Nat. Rev. Endocrinol..

[15] Haugen B.R., Alexander E.K., Bible K.C., Doherty G.M., Mandel S.J., Nikiforov Y.E., Pacini F., Randolph G.W., Sawka A.M., Schlumberger M. (2016). 2015 American Thyroid Association Management Guidelines for Adult Patients with Thyroid Nodules and Differentiated Thyroid Cancer: The American Thyroid Association Guidelines Task Force on Thyroid Nodules and Differentiated Thyroid Cancer. Thyroid.

[16] Totesora D., Chua-Agcaoili M.T. (2019). Cystic Papillary Thyroid Carcinoma: A Case Report. J. ASEAN Fed. Endocr. Soc..

[17] Donzuso G., Mostile G., Nicoletti A., Zappia M. (2019). Correction to: Basal ganglia calcifications (Fahr’s syndrome): Related conditions and clinical features. Neurol. Sci..

[18] Nardi F., Basolo F., Crescenzi A., Fadda G., Frasoldati A., Orlandi F., Palombini L., Papini E., Zini M., Pontecorvi A. (2014). Italian consensus for the classification and reporting of thyroid cytology. J. Endocrinol. Investig..

[19] Essig G.F., Porter K., Schneider D., Debora A., Lindsey S.C., Busonero G., Fineberg D., Fruci B., Boelaert K., Smit J.W. (2013). Fine needle aspiration and medullary thyroid carcinoma: The risk of inadequate preoperative evaluation and initial surgery when relying upon FNAB cytology alone. Endocr. Pract..

[20] Kihara M., Hirokawa M., Kudo T., Hayashi T., Yamamoto M., Masuoka H., Higashiyama T., Fukushima M., Ito Y., Miya A. (2018). Calcitonin measurement in fine-needle aspirate washout fluid by electrochemiluminescence immunoassay for thyroid tumors. Thyroid. Res..

[21] Diazzi C., Madeo B., Taliani E., Zirilli L., Romano S., Granata A.R., De Santis M.C., Simoni M., Cioni K., Carani C. (2013). The diagnostic value of calcitonin measurement in wash-out fluid from fine-needle aspiration of thyroid nodules in the diagnosis of medullary thyroid cancer. Endocr. Pract..

[22] Boi F., Maurelli I., Pinna G., Atzeni F., Piga M., Lai M.L., Mariotti S. (2007). Calcitonin measurement in wash-out fluid from fine needle aspiration of neck masses in patients with primary and metastatic medullary thyroid carcinoma. J. Clin. Endocrinol. Metab..

[23] Trimboli P., Giovanella L., Crescenzi A., Romanelli F., Valabrega S., Spriano G., Cremonini N., Guglielmi R., Papini E. (2014). Medullary thyroid cancer diagnosis: An appraisal. Head Neck.

[24] Faggiano A., Giannetta E., Modica R., Albertelli M., Barba L., Dolce P., Motta C., Deiana M.G., Martinelli R., Zamponi V. (2023). Calcium-stimulated calcitonin test for the diagnosis of medullary thyroid cancer: Results of a multicenter study and comparison between different assays. Minerva Endocrinol..

[25] Mian C., Perrino M., Colombo C., Cavedon E., Pennelli G., Ferrero S., De Leo S., Sarais C., Cacciatore C., Manfredi G.I. (2014). Refining calcium test for the diagnosis of medullary thyroid cancer: Cutoffs, procedures, and safety. J. Clin. Endocrinol. Metab..

[26] American Thyroid Association Guidelines Task F., Kloos R.T., Eng C., Evans D.B., Francis G.L., Gagel R.F., Gharib H., Moley J.F., Pacini F., Ringel M.D. (2009). Medullary thyroid cancer: Management guidelines of the American Thyroid Association. Thyroid.

[27] Toledo S.P., Lourenco D.M., Santos M.A., Tavares M.R., Toledo R.A., Correia-Deur J.E. (2009). Hypercalcitoninemia is not pathognomonic of medullary thyroid carcinoma. Clinics.

[28] Unluhizarci K., Akgun H., Oz B., Karaca Z., Tanriverdi F., Kelestimur F. (2017). Patients with papillary thyroid carcinoma associated with high stimulated serum calcitonin levels. Endocrinol. Diabetes Metab. Case Rep..

[29] Zwermann O., Piepkorn B., Engelbach M., Beyer J., Kann P. (2002). Abnormal pentagastrin response in a patient with pseudohypoparathyroidism. Exp. Clin. Endocrinol. Diabetes.

[30] Adachi I., Abe K., Tanaka M., Yamaguchi K., Miyakawa S. (1976). Plasma human calcitonin (hCT) levels in normal and pathologic conditions, and their responses to short calcium or tetragastrin infusion. Endocrinol. Jpn..

[31] Bastepe M., Lane A.H., Juppner H. (2001). Paternal uniparental isodisomy of chromosome 20q--and the resulting changes in GNAS1 methylation--as a plausible cause of pseudohypoparathyroidism. Am. J. Hum. Genet..

[32] Underbjerg L., Malmstroem S., Sikjaer T., Rejnmark L. (2018). Bone Status Among Patients With Nonsurgical Hypoparathyroidism, Autosomal Dominant Hypocalcaemia, and Pseudohypoparathyroidism: A Cohort Study. J. Bone Miner. Res..

[33] Brancatella A., Mantovani G., Elli F.M., Borsari S., Marcocci C., Cetani F. (2020). A severe inactivating PTH/PTHrP signaling disorder type 2 in a patient carrying a novel large deletion of the GNAS gene: A case report and review of the literature. Endocrine.

[34] Aliapoulios M.A., Voelkel E.F., Munson P.L. (1966). Assay of human thyroid glands for thyrocalcitonin activity. J. Clin. Endocrinol. Metab..

[35] Lee J.B., Tashjian A.H., Streeto J.M., Frantz A.G. (1968). Familial pseudohypoparathyroidism. Role of parathyroid hormone and thyrocalcitonin. N. Engl. J. Med..

[36] Suh S.M., Kooh S.W., Chan A.M., Fraser D., Tashjian A.H. (1969). Pseudohypoparathyroidism: No improvement following total thyroidectomy. J. Clin. Endocrinol. Metab..

[37] Deftos L.J., Powell D., Parthemore J.G., Potts J.T. (1973). Secretion of calcitonin in hypocalcemic states in man. J. Clin. Investig..

[38] Birkenhager J.C., Seldenrath H.J., Hackeng W.H., Schellekens A.P., van der Veer A.L., Roelfsema F. (1973). Calcium and phosphorus metabolism parathyroid hormone, calcitonin and bone histology in pseudohypoparathyroidism. Eur. J. Clin. Investig..

[39] Tessaris D., Bonino E., Weber G., Wasniewska M., Corica D., Pitea M., Scire G., Caruso-Nicoletti M., Fintini D., de Sanctis L. (2021). Pseudohypoparathyroidism: Application of the Italian common healthcare-pathway for a homogeneous clinical approach and a shared follow up. Ital. J. Pediatr..

[40] Nikiforov Y.E., Carty S.E., Chiosea S.I., Coyne C., Duvvuri U., Ferris R.L., Gooding W.E., LeBeau S.O., Ohori N.P., Seethala R.R. (2015). Impact of the Multi-Gene ThyroSeq Next-Generation Sequencing Assay on Cancer Diagnosis in Thyroid Nodules with Atypia of Undetermined Significance/Follicular Lesion of Undetermined Significance Cytology. Thyroid.

[41] Fortuna G.M.G., Rios P., Rivero A., Zuniga G., Dvir K., Pagacz M.M., Manzano A. (2020). Papillary Thyroid Carcinoma With Cystic Changes in a Patient With Prior History of Toxic Nodule. J. Investig. Med. High Impact. Case Rep..

[42] Yang G.C., Stern C.M., Messina A.V. (2010). Cystic papillary thyroid carcinoma in fine needle aspiration may represent a subset of the encapsulated variant in WHO classification. Diagn. Cytopathol..

[43] Elrick H., Albright F., Bartter F.C., Forbes A.P., Reeves J.D. (1950). Further studies on pseudo-hypoparathyroidism: Report of four new cases. Acta Endocrinol..

[44] Mann J.B., Alterman S., Hills A.G. (1962). Albright’s hereditary osteodystrophy comprising pseudohypoparathyroidism and pseudo-pseudohypoparathyroidism. With a report of two cases representing the complete syndrome occurring in successive generations. Ann. Intern. Med..

[45] Linglart A., Bastepe M., Juppner H. (2007). Similar clinical and laboratory findings in patients with symptomatic autosomal dominant and sporadic pseudohypoparathyroidism type Ib despite different epigenetic changes at the GNAS locus. Clin. Endocrinol..

[46] Dedivitis R.A., Aires F.T., Cernea C.R. (2017). Hypoparathyroidism after thyroidectomy: Prevention, assessment and management. Curr. Opin. Otolaryngol. Head Neck Surg..

